# Mathematical Modeling of *Streptococcus pneumoniae* Colonization, Invasive Infection and Treatment

**DOI:** 10.3389/fphys.2017.00115

**Published:** 2017-03-02

**Authors:** Elisa Domínguez-Hüttinger, Neville J. Boon, Thomas B. Clarke, Reiko J. Tanaka

**Affiliations:** ^1^Department of Bioengineering, Imperial College LondonLondon, UK; ^2^Instituto de Ecología, Universidad Nacional Autónoma de MéxicoMexico City, Mexico; ^3^Department of Medicine, Imperial College LondonLondon, UK

**Keywords:** *Streptococcus pneumoniae*, upper airway epithelium, data integration, hybrid systems, commensal bacteria, systems biology, antibiotics resistance

## Abstract

*Streptococcus pneumoniae* (*Sp*) is a commensal bacterium that normally resides on the upper airway epithelium without causing infection. However, factors such as co-infection with influenza virus can impair the complex *Sp*-host interactions and the subsequent development of many life-threatening infectious and inflammatory diseases, including pneumonia, meningitis or even sepsis. With the increased threat of *Sp* infection due to the emergence of new antibiotic resistant *Sp* strains, there is an urgent need for better treatment strategies that effectively prevent progression of disease triggered by *Sp* infection, minimizing the use of antibiotics. The complexity of the host-pathogen interactions has left the full understanding of underlying mechanisms of *Sp*-triggered pathogenesis as a challenge, despite its critical importance in the identification of effective treatments. To achieve a systems-level and quantitative understanding of the complex and dynamically-changing host-*Sp* interactions, here we developed a mechanistic mathematical model describing dynamic interplays between *Sp*, immune cells, and epithelial tissues, where the host-pathogen interactions initiate. The model serves as a mathematical framework that coherently explains various *in vitro* and *in vitro* studies, to which the model parameters were fitted. Our model simulations reproduced the robust homeostatic *Sp*-host interaction, as well as three qualitatively different pathogenic behaviors: immunological scarring, invasive infection and their combination. Parameter sensitivity and bifurcation analyses of the model identified the processes that are responsible for qualitative transitions from healthy to such pathological behaviors. Our model also predicted that the onset of invasive infection occurs within less than 2 days from transient *Sp* challenges. This prediction provides arguments in favor of the use of vaccinations, since adaptive immune responses cannot be developed *de novo* in such a short time. We further designed optimal treatment strategies, with minimal strengths and minimal durations of antibiotics, for each of the three pathogenic behaviors distinguished by our model. The proposed mathematical framework will help to design better disease management strategies and new diagnostic markers that can be used to inform the most appropriate patient-specific treatment options.

## 1. Introduction

*Streptococcus pneumoniae* (*Sp*) is a commensal bacterium that is part of the upper airway microbiota. While it normally resides on the upper airway epithelium without causing serious infection or tissue damaging inflammation (World Health Organization, 2012), factors such as co-infection with the influenza virus often result in the development of life-threatening infectious and inflammatory diseases, including pneumonia, meningitis or even sepsis (World Health Organization, 2012; McCullers, 2014), since these factors can cause a weakened immune response to *Sp* or tissue damage that may disrupt the normal interactions between *Sp* and host. The threat of *Sp* infection has been increasing despite interventions by widely available antibiotics, due to the increasing presence of multiple antibiotic-resistant *Sp* strains (Nuorti et al., 1998; McCullers et al., 2000). Reduced susceptibility to penicillin was detected in all WHO regions (World Health Organization, 2014) and the pneumococcus remains a major cause of morbidity and mortality, not solely from the lower lung infection Siegel and Weiser (2015). There is an urgent need to devise better intervention strategies that can effectively halt the onset or persistence of *Sp*-mediated pathology at its early stages using a minimal amount of antibiotics for a short duration, in order to avoid the emergence of further antibiotic-resistant *Sp* strains (Schrag et al., 2001; Prina et al., 2015b).

Identification and design of effective intervention strategies require systems-level and quantitative understanding of the complex and dynamically-changing host-pathogen interactions that can lead to either healthy *Sp* colonization or pathological conditions, such as infection or inflammation. This paper proposes a mathematical model of the host-pathogen interactions between *Sp* and the upper airway epithelium, the initial site of interaction between *Sp* and the host which is the first step in all disease tiggered by this bacterium (Siegel and Weiser, 2015). We analyse the model to systematically and quantitatively investigate the mechanisms by which the homeostatic interactions are disrupted, for example by a weakened barrier function (McCullers, 2014) or immune suppression (Didierlaurent et al., 2008), and cause the onset of infectious processes.

Previously proposed mathematical models (Smith et al., 2011; Shrestha et al., 2013; Smith et al., 2013; Mochan et al., 2014) considered *Sp* infections in the lung which is a normally sterile site of the airway epithelium. In this paper, we develop a mechanistic model of homeostatic interactions between the host's upper airway and *Sp* as a commensal bacterium, based on a variety of experimental data from *in vivo* and *in vitro* studies. Given that the tissue-damaging effects of neutrophil transmigration are responsible for part of the pathology of infection (Chin and Parkos, 2007; Zemans et al., 2009), we specifically model how impaired host-pathogen interactions lead to loss of epithelial homeostasis and serious infection. Our mechanistic model describes dynamic interplays between *Sp*, immune cells, and epithelial tissues by a hybrid system of ordinary differential equations (ODEs), and elucidates the mechanisms by which commensal bacteria cause infection.

Our model demonstrates a robust behavior of healthy clearance of asymptomatic pneumococcal colonization without overt disease. Perturbation of the model parameters, corresponding to virtual patient cohorts, demonstrates three clinically observed pathological behaviors (disease phenotypes) triggered by disrupted *Sp*-host interactions. Using this mathematical model of pneumococcal colonization, we further suggest optimal treatment regimens that minimize use of antibiotics to intervene the pathogenic processes for each patient cohort. As colonization is a prerequisite for all pneumococcal disease (Siegel and Weiser, 2015), studying and the modeling of colonization by *Sp* to understand its interaction with the host could be important for not only looking at invasive infections but also other types of interaction/infection of the pneumococcus and host.

## 2. Results

### 2.1. Mathematical model of *Sp* colonization in the upper airway epithelium

**Figure 1:**
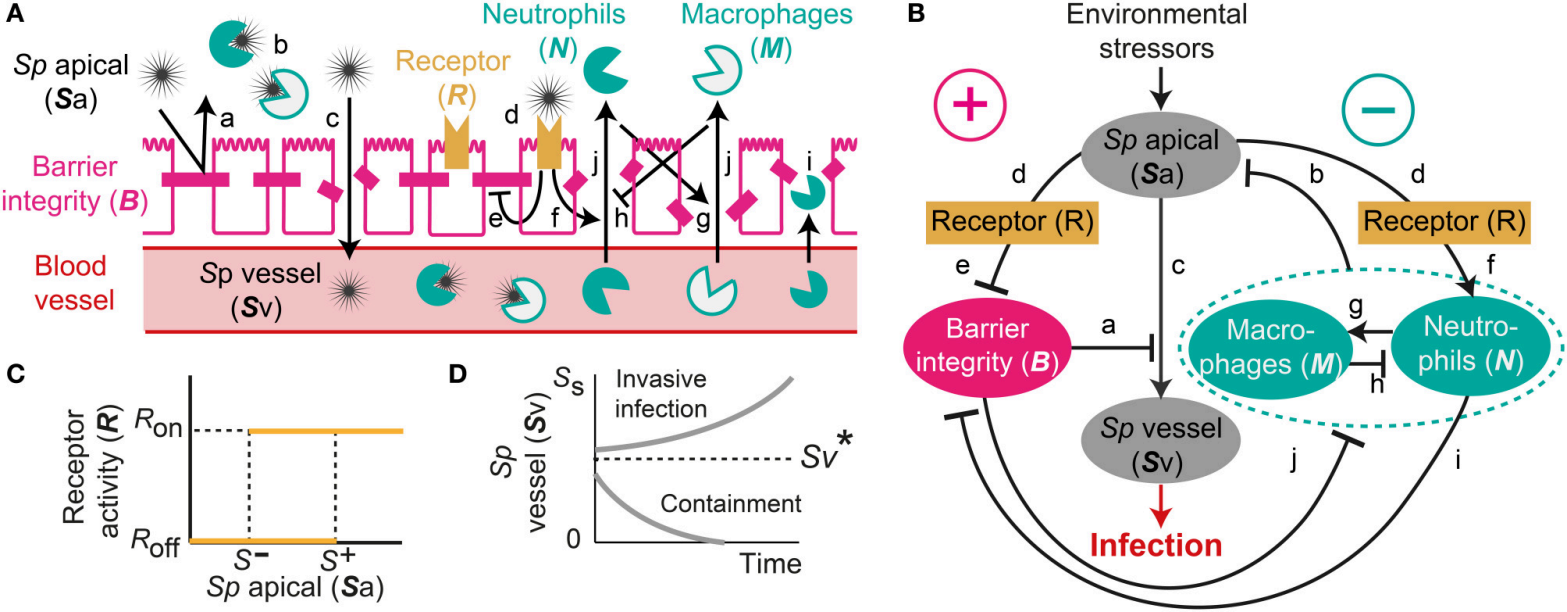
**A mechanistic model of ***Sp***. colonization. (A)** A schematic diagram of the processes included in the model. **(B)** The dynamic interplay between environmental stressors, barrier function and immune responses regulates infiltration of *Sp* to the blood vessel, which can result in infection. **(C)** *R*-switch for reversible activation of TLRs. **(D)** *S*_v_-switch for the threshold behavior (invasive infection or containment) of the infiltrated *Sp* in the blood vessel.

Our proposed mathematical model of *Sp* colonization (Figures 1A,B) is a system-level representation of the prominent interactions between *Sp*, the airway epithelium, and immune cells (a–j) in Figures 1A,B, that were identified based on the empirical evidence from numerous experimental *in vivo* and *in vitro* studies as detailed below.

Under homeostatic conditions, a population of commensal bacteria, *Sp*, resides in the lumen on the apical side of the airway epithelium, where they are contained by a competent epithelial barrier integrity (Beisswenger et al., 2007) (Figures 1Aa,Ba) and immune responses mediated by neutrophils and macrophages (Dick et al., 2008; Standish and Weiser, 2009) (Figures 1Ab,Bb). Through disrupted barrier, apically located *Sp* can translocate to reach the blood vessel (Beisswenger et al., 2007) (Figures 1Ac,Bc), where they are either killed by resident immune cells that circulate in the blood (Li et al., 2002; Li, 2004), or grow uncontrollably and result in invasive infection if the immune cells cannot contain the translocated *Sp* (Silverstein and Rabadan, 2012). The amount of the translocated *Sp* in the blood vessel is therefore a determinant of whether disrupted *Sp*-host interactions cause serious infection such as sepsis.

Translocation of *Sp* occurs through the airway epithelial barrier, whose integrity is regulated by the apically located bacteria load. The bacteria bind to Pattern-Recognition immune receptors, specifically Toll-like receptors (TLR2s) that are preferentially expressed on the apical side of the airway epithelial cells (Melkamu et al., 2009), and activate the TLR signaling cascade (Figures 1Ad,Bd). The activation of the TLR cascade in epithelial cells decrease the barrier integrity of the airway epithelium (Figures 1Ae,Be) by TLR-mediated activation of proteases that damage the epithelial cells (Oggioni et al., 2004; Schmeck et al., 2004; Attali et al., 2008; Tieu et al., 2009) and by reduction of the barrier recovery rate due to the increased expression of the transcriptional repressor SNAIL1, which inhibits the expression of *claudin*, a component of the tight junctions (Clarke et al., 2011).

Active TLR signaling also induces recruitment of neutrophils from the neutrophil pool in the blood vessel, via the release of IL-17 (Zhang et al., 2009) that activates neutrophil-attracting interleukins IL-8 (Lindén, 2001) (Figures 1Af,Bf). The recruited neutrophils trigger transmigration of macrophages to the site of infection (Zhang et al., 2009), further potentiating the immune responses to the apically located pathogens (Figures 1Ag,Bg), whereas macrophages on the lumen restrict neutrophil transmigration (Zhang et al., 2009) by releasing neutrophil-repellent anti-inflammatory cytokines (Knapp et al., 2003) (Figures 1Ah,Bh). Transmigrating neutrophils release barrier degrading proteases (Chin et al., 2008) to reduce the barrier integrity (Nash et al., 1987; Nusrat et al., 1997; Zemans et al., 2009) (Figures 1Ai,Bi). The reduced barrier integrity in turn allows more transmigration of both neutrophils and macrophages from the blood vessel to the site of infection (Nash et al., 1987) (Figures 1Aj,Bj).

The model elucidates the main control structure of the system that maintains homeostatic interactions between commensal bacteria, *Sp*, and the host, via a dynamic interplay between environmental stressors, epithelial barrier integrity and immune responses (Figure 1B). At the apical side of the airway epithelium, *Sp* load is regulated via activation of TLRs, which induce immune responses that decrease the bacterial load but also reduce the epithelial barrier integrity. While the reduced barrier integrity enables transmigration of immune cells from the blood vessel for effective killing of *Sp* at the apical side of the epithelium barrier, it also allows transmigration of *Sp* from the apical side of the epithelium to the blood vessel, potentially causing systemic infection (sepsis). The dynamic interplay between the immune responses and the epithelial barrier integrity are further modulated by their mutual inhibition.

Our model further assumes two switches, an *R*-switch for TLR activation and an *S*_v_-switch for the growth of the transmigrated bacteria in the blood vessel, based on the experimental evidence described below. The *R*-switch for *Sp*-mediated activation of TLRs reflects the observations that low concentrations of *Sp* do not cause activation of TLR signaling, while high concentrations lead to a sharp increase in TLR activity with hysteresis (He et al., 2009; Shalek et al., 2013; Sung et al., 2014). We model the *R*-switch by a perfect switch, which is a phenomenological representation of the bistable switch (Sung et al., 2014), and is described by the off- and on-states (*R* = *R*_off_ and *R*_on_) with the activation (*S*^+^) and inactivation (*S*^−^) thresholds for the critical concentrations of apically located *Sp* that abruptly and sharply turn on-or-off TLR activity (Figure 1C and Equation 2). The *S*_v_-switch reflects the observations that transmigrated bacteria in the blood vessel (*S*_v_) either overgrow (Benton et al., 1997) or are contained by resident immune cells depending on the bacterial concentration (Supplementary Figure 3B). We model the *S*_v_-switch with a switching threshold of $S_{\text{v}}^{*}$, above which the infiltrated bacteria in the blood vessel grow exponentially (Figure 1D).

The resulting model is described by a hybrid system of five ODEs (Equation 1). The nominal values of the 24 model parameters (Table 1) were derived by fitting the model outcome to datasets from 11 independent studies, namely three *in vivo* studies (Benton et al., 1997; Zhang et al., 2009 and our own experiment) and eight *in vitro* studies (Nash et al., 1987; Coyne et al., 2002; Lagrou et al., 2003; Attali et al., 2008; Chin et al., 2008; Komori et al., 2011; Hathaway et al., 2012; Kwok et al., 2012), as detailed in the Supplementary Material. Our model therefore provides a coherent mathematical framework to explain both *in vivo* and *in vitro* data.

**Table 1:** **Nominal parameters of the model**.

**Parameter**	**Description**	**Value**	**References**
*N*_v_	Size of the neutrophil pool	10^8^	Tanaka et al., 2015
δ_N_	*N* degradation rate	6.1 × 10^−2^/h	Tanaka et al., 2015
κ_B_	Barrier recovery rate	4.6 × 10^−2^/h	Coyne et al., 2002
κ_S_	Bacteria growth rate	4.8 × 10^−1^/h	Hathaway et al., 2012
$\tilde{B}$	Nominal barrier integrity	1	
*S*^+^	Activation threshold for *R*-switch	10^7^ CFU/ml	Komori et al., 2011; Kwok et al., 2012
*S*^−^	Deactivation threshold for *R*-switch	10^3^ CFU/ml	Komori et al., 2011; Kwok et al., 2012
θ_S_	Rate of bacterial transmigration through barrier	1.1 × 10^−4^/h	Lagrou et al., 2003; Attali et al., 2008; Zhang et al., 2009
ϵ_SB_	Inhibition rate of *S*_a_ transmigration by *B*	3.1	Lagrou et al., 2003; Attali et al., 2008; Zhang et al., 2009
ϵ_BS_	Inhibition rate of *B* recovery by *S*_a_	2.6 × 10 ml/CFU	Lagrou et al., 2003; Attali et al., 2008
ϕ_SB_	Degradation rate of *B* by *S*_a_	1.4 × 10^−1^ml/CFU × h	Lagrou et al., 2003; Attali et al., 2008
ϵ_NB_	Inhibition Rate of *N* recruitment by *B*	3.6 × 10	Nash et al., 1987; Chin et al., 2008
ϵ_MB_	Inhibition rate of *M* recruitment by *B*	= ϵ_NB_	Nash et al., 1987; Chin et al., 2008
ϕ_NB_	Degradation rate of *B* by *N*	4.0 × 10^−8^ ml/cells × h	Nash et al., 1987; Chin et al., 2008
μ_S_	Saturation limit for *S*_a_	3.7 × 10^4^ CFU/ml	Zhang et al., 2009
ϕ_NS_	Rate of *S*_a_ killing by *N*	6.1 × 10^−4^ ml/cells × h	Zhang et al., 2009
ϕ_MS_	Rate of *S*_a_ killing by *M*	6.3 × 10^−3^ ml/cells × h	Zhang et al., 2009
*K*	Half-killing constant of *S*_v_	1.3 × 10^4^ CFU/ml	Benton et al., 1997 and Figure S3
δ_S_	Rate of *S*_v_ killing by circulating immune cells	6.9 × 10^3^ cells/ml × h	Benton et al., 1997 and Figure S3
α	Rate of *N* recruitment by *S*_a_	0.465 × 150*x*10^(−8)^ ml/CFU × h	Zhang et al., 2009
ϵ_NM_	Inhibition rate of *N* recruitment by *M*	1.6 × 10^−1^ ml/cells	Zhang et al., 2009
β	Rate of *M* recruitment by *N*	2.6 × 10^−2^ ml/cells × h	Zhang et al., 2009
*Mv*	Number of macrophage pool	3.0 × 10^−1^ cells/ml	Zhang et al., 2009
δ_M_	*M* degradation rate	6.4 × 10^−5^/h	Zhang et al., 2009

### 2.2. Healthy clearance of asymptomatic *Sp* colonization is robustly observed

**Figure 2:**
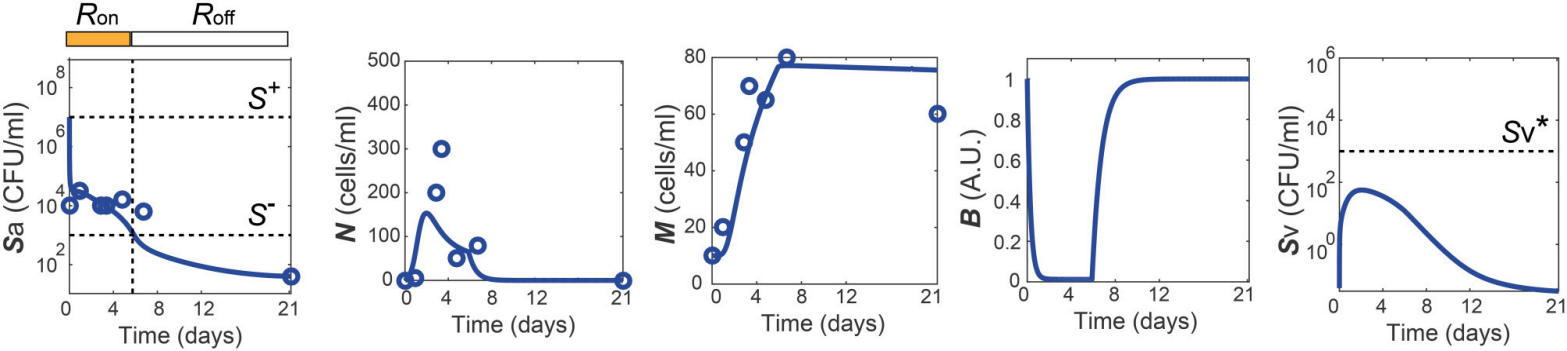
**Healthy recovery from a transient ***Sp*** challenge**. Blue circles and solid lines represent the *in vivo* experimental data from Zhang et al. (2009) and the model prediction, respectively.

One of the dataset used for the parameter estimation was obtained from *in vivo* studies in Zhang et al. (2009), where the mice were challenged with 10^7^ CFU of *Sp* and recovered their healthy state, which is characterized by nonzero apical commensal bacterial load that does not trigger host responses. Our model was fitted to reproduce the experimental measurement in Zhang et al. (2009) for the apical bacterial load (*S*_a_) and the concentrations of neutrophils (*N*) and macrophages (*M*) (Figure 2A). Both the experimental data and our model simulation demonstrate that the transient *Sp* challenge (increase of *S*_a_) triggers a transient increase in *N* and a subsequent increase in *M*. These immune responses can bring *S*_a_ down to a homeostatic level, when the saturation limit for *S*_a_ is not high enough, which enabled the recovery of the mice from the bacterial challenge within 7 days without demonstrating invasive infection.

The simulation of our model with the data-calibrated nominal parameters further predicts the dynamics of three variables that were not measured in this experiment, TLR activity (*R*), barrier integrity (*B*) and infiltrated *Sp* in the blood vessel (*S*_v_), thereby explaining the underlying mechanism of the healthy recovery from a bacterial challenge. Upon a *Sp* challenge, the apically located bacterial load becomes high enough ($S_{\text{a}}\left(0\right)>S^{+}$) to activate TLRs (*R* = *R*_on_), which trigger recruitment of immune cells to the site of infection. These immune responses bring the initially high *S*_a_ down to below *S*^−^, where the *R*-switch turns off (*R* = *R*_off_) and stays off as *S*_a_ remains below *S*^+^, as suggested by the focal point analysis (see Methods). The epithelial barrier integrity (*B*) continuously decreases while the *R*-switch is on (*R* = *R*_on_), allowing bacteria to invade the blood vessel, as demonstrated by a rise in *S*_v_. However, a healthy clearance of *S*_v_ is achieved without causing sepsis, since the peak of *S*_v_ remains below the threshold, $S_{\text{v}}^{*}$, of the *S*_v_-switch. Note that both the experiments and our model simulation demonstrate that *M* stays high while *B* is kept high after *N* goes to zero, suggesting the importance of *M* as an immune mediator that does not compromise the barrier integrity.

**Figure 3:**
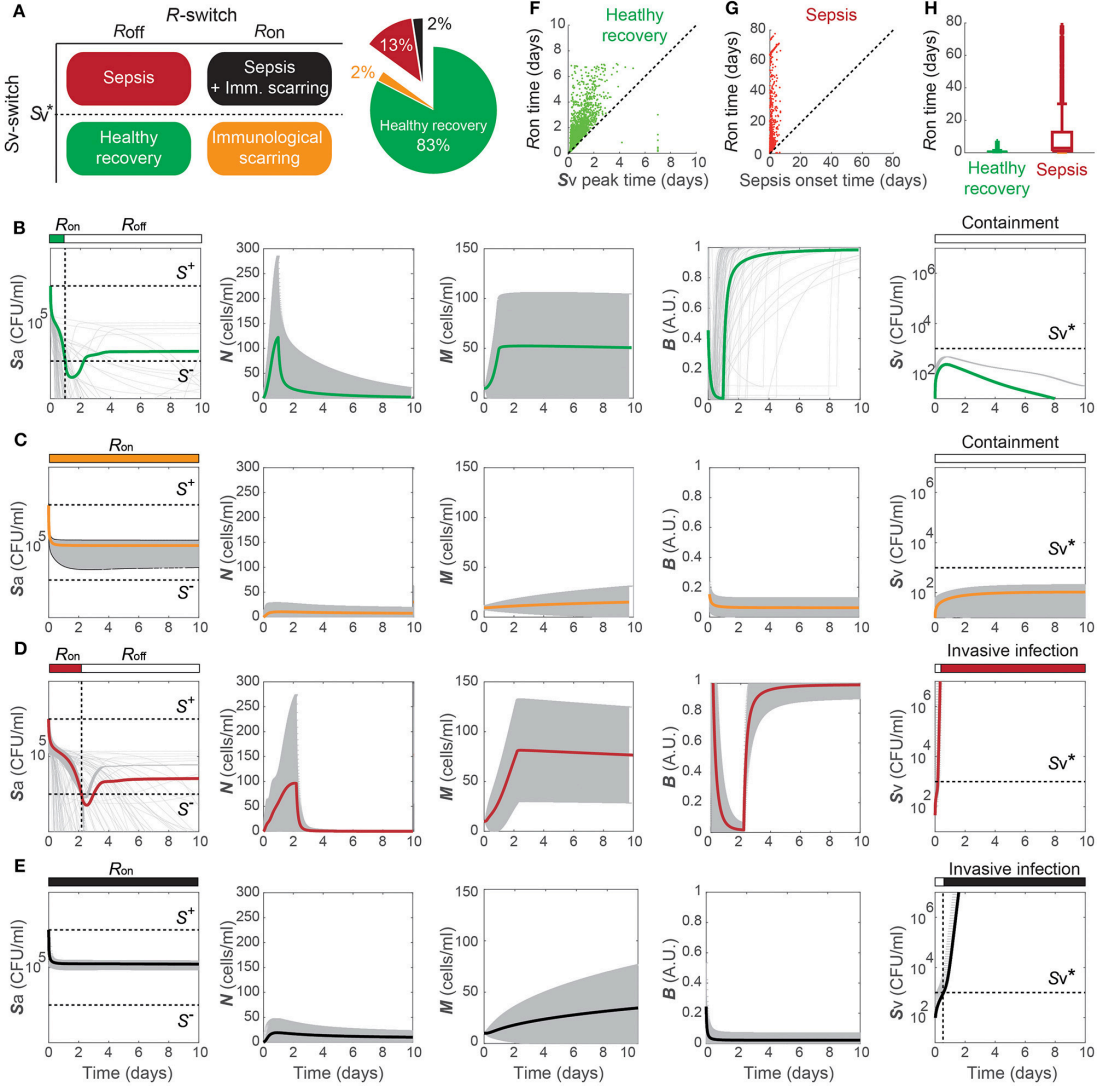
**Four phenotypes resulting from alterations in the ***Sp***-host interactions. (A)** Four (a healthy and three pathological) phenotypes determined by the states of *R*- and *S*_v_-switches, and their respective frequency of observation in 10, 000 simulations with varying parameters for our model. The states of the *R*- and *S*_v_-switches determine whether host response persists causing immunological scarring, and whether sepsis occurs, respectively. **(B–E)** The dynamics of the four phenotypes: healthy recovery **(B)**, immunological scarring **(C)**, sepsis **(D)** and sepsis accompanied with scarring **(E)**. Solid lines and the gray shaded regions correspond to the mean dynamics and the ± standard deviation. **(F)** *R*-switch ON time vs. Time to reach the peak of *S*_v_ for the healthy recovery case. **(G)** *R*-switch ON time vs. Time for sepsis onset for the sepsis phenotype. **(H)** Boxplots representing the minimum, first quartile, median, third quartile, maximum and outlayers of the *R*-switch ON time for healthy recovery and sepsis cases.

The healthy recovery behavior described above is characterized in our model by convergence to the off-state of TLR activity (*R* = *R*_off_) accompanied by the containment of *S*_v_ $\left(S_{\text{v}}<S_{\text{v}}^{*}\right)$, and is robustly observed under perturbations to the parameter values. Among 10,000 simulations conducted by randomly sampling parameter values from an uniform distribution over two orders of magnitude around the nominal values, 83% of the simulations demonstrated a healthy recovery from a transient *Sp* challenge (Figures 3A,B). In 98% of the healthy recovery cases computationally observed, *S*_v_ reached its peak while *R* = *R*_on_ (Figure 3E), suggesting that the appropriate host responses via TLR activation are responsible for containing *S*_v_. The appropriate level of the barrier damage by active TLRs enables effective recruitment of immune cells that can reduce *S*_a_, but prevents excessive transmigration of *S*_a_ to *S*_v_, keeping the *S*_v_ lower than the threshold, $S_{\text{v}}^{*}$. The robust appearance of the healthy recovery in our model simulations confirms that that our model can coherently explain the mechanism behind the healthy recovery of the host from pneumococcal colonization, which can be effectively cleared by the natural host responses without any treatments.

### 2.3. Four phenotypes classified by the double switch

The remaining 17% of the simulations with parameters perturbed from their nominal values demonstrated systems dynamics that correspond to serious infection or inflammation. They are classified into three disease phenotypes, depending on the states of the *S*_v_- and *R*-switches (Figure 3A). The state of the *S*_v_-switch determines whether sepsis occurs due to invasive infection of $S_{\text{v}}\left(>S_{\text{v}}^{*}\right)$, and that of the *R*-switch determines whether *immunological scarring* occurs due to persistent host responses caused by *R* = *R*_on_. Immunological scarring refers to the cumulative and long-term effects of immune response to pathogens, including tissue remodeling and altered immune responses to new pathogenic challenges, that persist after the pathogenic organism has been cleared (Fonseca et al., 2015). In our simulations, 13% demonstrated sepsis without immunological scarring ($S_{\text{v}}>S_{\text{v}}^{*}$ and *R* = *R*_off_) and the other two disease phenotypes, immunological scarring (*R* = *R*_on_) with and without sepsis, were observed 2% each (Figure 3A).

Immunological scarring is characterized by a persistent on-state of the *R*-switch due to *S*_a_ staying above *S*^−^ (Figure 3C). The *R*-switch triggers persistent host responses leading to sustained immune responses which are not strong enough to decrease *S*_a_ below *S*^−^ but cause persistent barrier damage. Note that the peak of *N* is much lower in the immunological scarring phenotype than that in the healthy recovery and sepsis phenotypes with *R* = *R*_off_ (Figures 3B,D), resulting in weak immune responses that are not sufficient to decrease *S*_a_. As a result, the host becomes vulnerable to a second bacterial attack due to the damaged barrier and the sub-threshold concentration of *S*_v_ $\left(<S_{\text{v}}^{*}\right)$, which stay as silent remainders of the first pathogenic challenge.

Sepsis is characterized by outgrowth of *S*_v_ once it surpasses the threshold $S_{\text{v}}^{*}$ (Figure 3C). In 99.7% of the sepsis phenotypes simulated by our model, the onset of sepsis occurs (when $S_{\text{v}}=S_{\text{v}}^{*}$ is achieved) while *R* is on (Figure 3G), suggesting that whether sepsis occurs or not is determined by the dynamics of *S*_v_ while *R* is on. It is similar with the healthy recovery case, where *S*_v_ reaches its peak below the threshold $S_{\text{v}}^{*}$, while *R* is on. Moreover, the duration of *R* = *R*_on_ is much longer for the sepsis phenotype compared to the healthy recovery phenotype (Figure 3H), suggesting that persistent host response may allow excessive transmigration of *Sp* into the blood vessel above $S_{\text{v}}^{*}$.

When both the *S*_v_- and *R*-switches are on, sepsis is accompanied by immunological scarring (Figure 3E), where the barrier is severely damaged and *S*_v_ continues increasing above $S_{\text{v}}^{*}$, while *S*_a_ remains above *S*^−^.

The four phenotypes, including a healthy phenotype and three disease phenotypes, correspond to different patient cohorts observed in the clinic. *Healthy recovery from colonization* is the most common outcome of host-pneumococcal interactions (Austrian, 1986) and corresponds to patients who can clear their symptoms from transient infection without any antibiotics treatment. *Sepsis* corresponds to patients who would develop systemic infection as a consequence of dysregulated transepithelial crossing of bacteria if no treatment is applied (Clarke et al., 2011; Siegel and Weiser, 2015). *Immunological scarring* corresponds to tissue-damaging inflammation that prevails even after clearance of the pathogens (Periselneris et al., 2015). The long-term deleterious consequences of such sterile inflammation and the associated tissue restructuring/damage are considered to underlie many diseases, including pulmonary fibrosis associated to previous *Sp* infections (Knippenberg et al., 2015), chronic obstructive pulmonary disease (Garcha et al., 2012) and cancer (Elinav et al., 2013; Pradere et al., 2014). A sustained activation TLR is recognized to be an important molecular player responsible for this tissue damage (Pradere et al., 2014), as in our model. *Sepsis with immunological scarring* corresponds to patient cohorts who would develop a severe infection with long-term deleterious effects in absence of treatment (Leibovici, 2013).

### 2.4. Risk factors for disease phenotypes

To identify the model parameters that affect the states of the *R*- and *S*_v_-switches thereby determine the four phenotypes, we conducted the global parameter sensitivity analysis of our model with respect to *R* and *S*_v_, respectively, using both Sobol and eFAST sensitivity indices (Marino et al., 2008; Cannavó, 2012).

**Figure 4:**
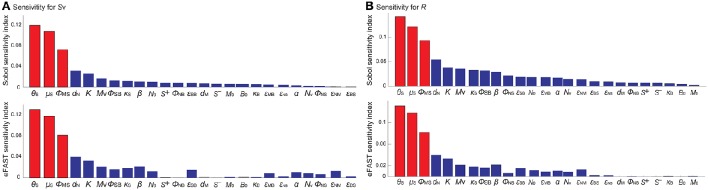
**Global sensitivity analysis of the model with respect to (A)** *S*_v_ and **(B)** *R*, using the SOBOL and eFAST sensitivity indices.

**Figure 5:**
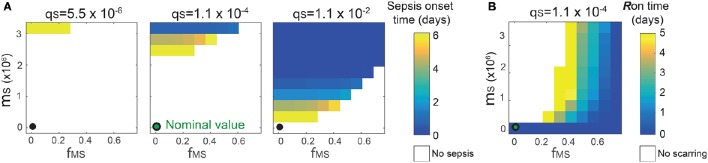
**Combinatorial effects of the three most sensitive parameters (θ_**S**_, μ_**S**_ and ϕ_**MS**_) on the sepsis onset time (A)** and the *R*_on_ time **(B)**. The black circles correspond to the nominal values for (μ_*S*_, ϕ_*MS*_) and the nominal value for θ_*S*_ is 1.1 × 10^−4^. Changes in θ_S_ do not affect the *R*_on_ time.

The analysis identified the three most sensitive parameters for the propensity to turn on both the *R*-switch (to develop immunological scarring) and the *S*_v_-switch (to develop sepsis) (Figure 4): the rate of bacterial transmigration through the barrier (θ_S_), the bacterial carrying capacity (μ_S_), and the killing rate of bacteria by macrophages (ϕ_MS_) further confirming the importance of macrophages. Simulations with systematic variations of these three parameters further suggest that they affect the occurrence of sepsis and of immunological scarring, as well as how quickly these occur after the *Sp* challenge (Figure 5).

These three sensitive parameters have a direct correspondence with risk factors for disease triggered by *Sp* that have been reported in the experimental literature. For example, increase in θ_S_ can be caused by co-infection, which damages the barrier directly or by having triggered previous immune responses (McCullers, 2014). Increase in μ_S_ is caused by previous infections, for example by influenza virus, that damage the tissue, increase nutrient contents (Siegel et al., 2014), or shift the microbiome composition affecting the dynamics of the different bacterial populations (McCullers, 2014). ϕ_MS_ can be affected for example by severe asthma (Liang et al., 2014).

Other parameters that are also affected by co-infection were not identified to be very sensitive for the propensity to develop sepsis (increase in *S*_v_) or unresolved host responses (increase in *R*) (Figure 4). These parameters include *S*^+^ which can increase as a consequence of TLR2 desensitization caused by a previous influenza virus infection (Didierlaurent et al., 2008), *M* that may increase as a consequence of previous infectious events (La Gruta et al., 2007; Yin et al., 2013), and the size of the neutrophil pool (*N*_v_) which may decrease by chemotherapy or severe infections (Dick et al., 2008). The unsensitivity to the initial conditions can be partially explained by the existence of a unique stable steady state corresponding to the healthy recovery.

### 2.5. A rapid onset of sepsis triggered by a transient *Sp* challenge

**Figure 6:**
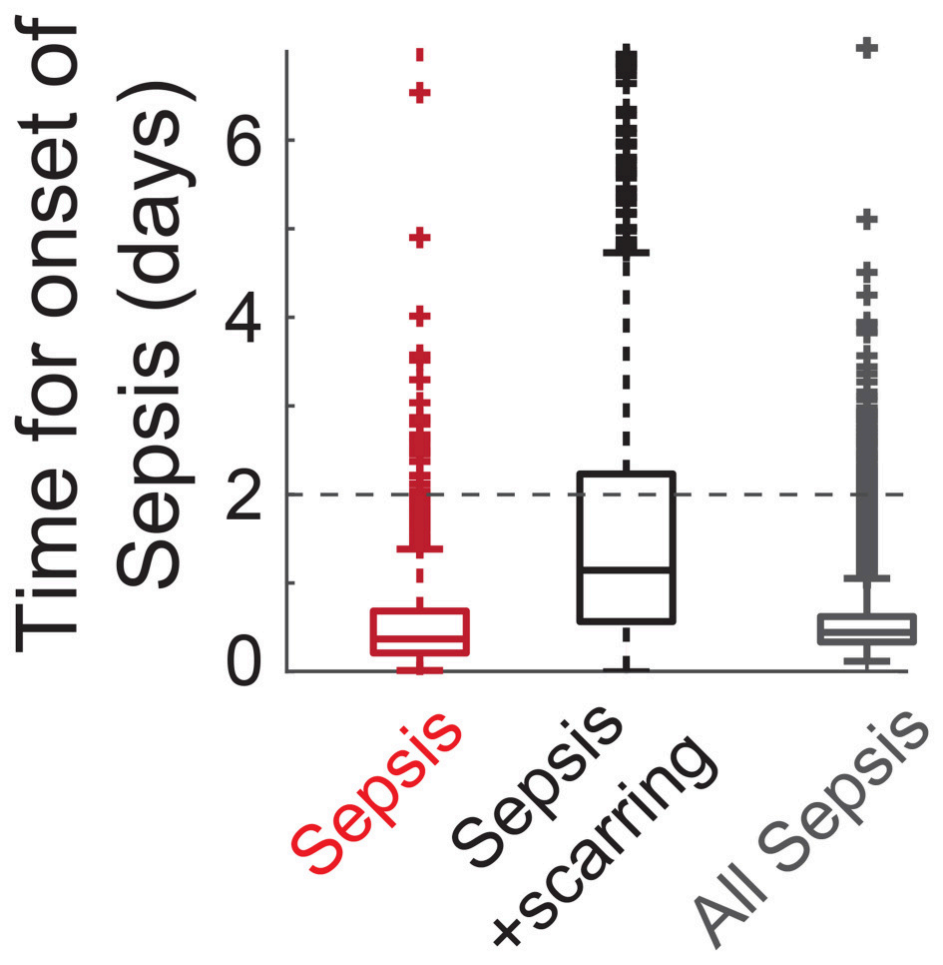
**Computationally predicted time for onset of sepsis**.

In the septic behavior observed in 15% of the simulations (Figures 3C,D), the sepsis occurred (*S*_v_ increases above $S_{\text{v}}^{*}$) within 2 days post *Sp* challenge in 79% of the cases (Figure 6). When sepsis is accompanied with immunological scarring (*R* stays on and *S*_v_ increases above $S_{\text{v}}^{*}$, Figure 3D), the time to sepsis is longer than when it is not (Figure 6). The computationally predicted rapid onset of the sepsis is consistent with experimental observations in Andonegui et al. (2009) that the mice either survived or died within 36 h upon *Sp* challenge applied directly into the lumen of the lungs. The results suggest that rapid treatments within 36 h are crucial to prevent the onset of sepsis.

Increasing the immune activity, for example by activation of adaptive immune responses, could be an effective way to decrease the risk of sepsis onset, as it elevates the switching threshold, $S_{\text{v}}^{*}$, which depends on the strength of the resident immune cells. While the adaptive immunity could be activated naturally, the time for activation of the adaptive immune responses (which involves the *de novo* differentiation of naive T cells into mature T cells) by infiltrated pathogens was experimentally evaluated to be more than 2 days in mice (Zheng and Flavell, 1997). Such slow activation of the adaptive immunity therefore cannot prevent the onset of sepsis within 36 h.

These results suggest that prophylactic activation of the adaptive immune responses, for example by vaccinations, could be an effective strategy to prevent the incidence of sepsis, as demonstrated by the protective effects of *Sp* vaccination in mice (Cao et al., 2013). It is also consistent with the clinical suggestions to use vaccines as a preventive measurement against transient bacterial challenges in the high-risk patients (World Health Organization, 2012).

### 2.6. Optimal antibiotics treatment regimens for each of the three patient cohorts

Using the proposed model, we investigate optimal treatment regimens and determine the minimal strength and duration of antibiotics treatment that are required to prevent or revert the pathological consequences of a transient *Sp* challenge. The minimal use of antibiotics is important for tackling the problem of antibiotics resistance (Nuorti et al., 1998), since the emergence of antibiotic-resistant *Sp* strains has been associated to the excessive use of antibiotics (Schrag et al., 2001; Prina et al., 2015b). We consider two different types of bactricidal antibiotics treatments in our modeling framework: apical application of antibiotics in the luminal side of the mucosa that decreases *S*_a_ and can thereby turn off the *R*-switch and stop the immunological scarring, and systemic application of antibiotics in the blood vessel that directly decreases *S*_v_ to prevent the onset of invasive infection (described in the Methods Section 4.3).

**Figure 7:**
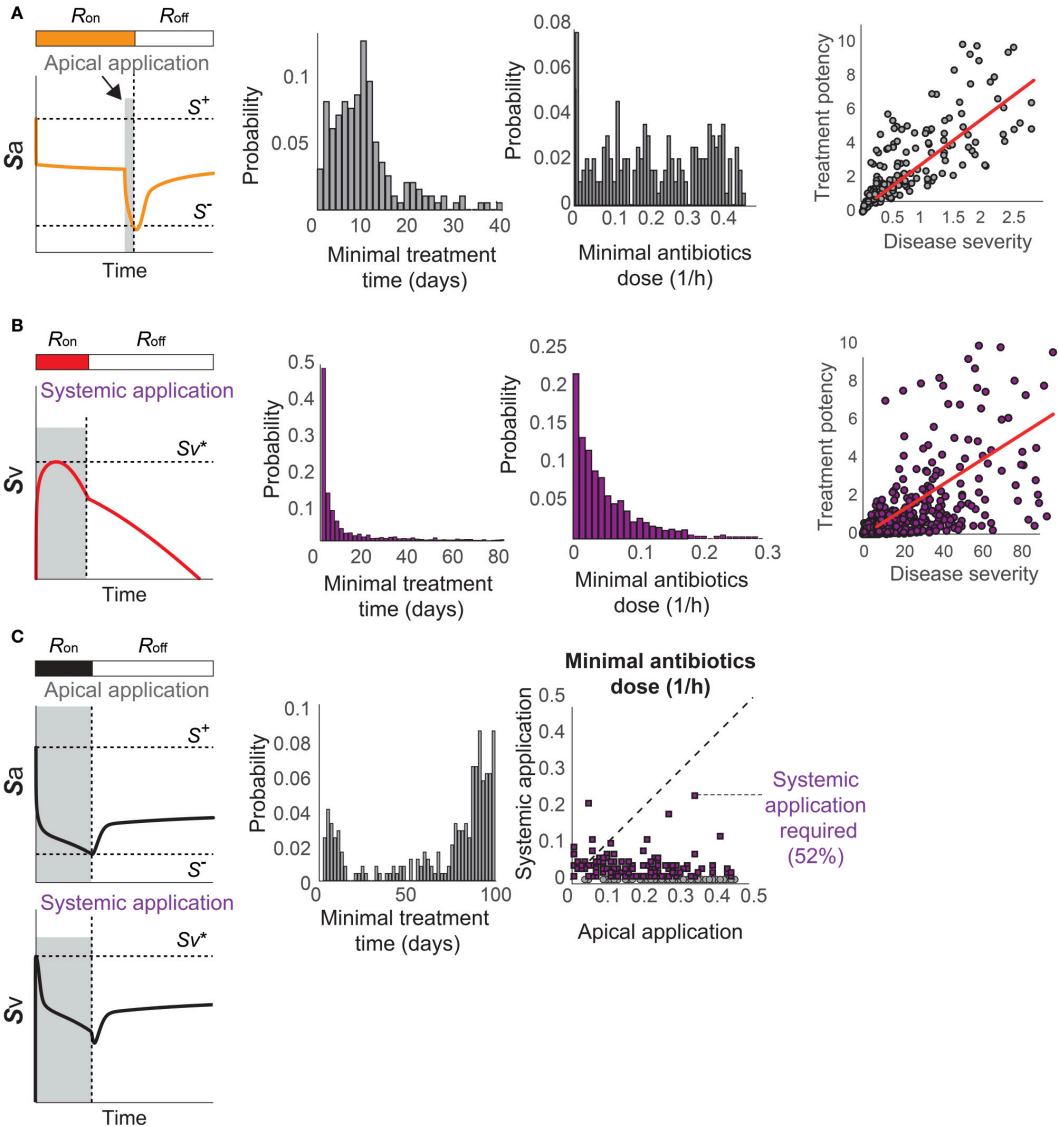
**Optimal antibiotics treatment strategies for patients with (A)** Immunological scarring to turn off the *R*-switch, **(B)** sepsis, and **(C)** sepsis and immunological scarring. Gray shaded regions in the left column denote the minimal time of application of antibiotics in the apical side of the epithelium or in the blood vessel (systemic application).

When the patients have immunological scarring without sepsis (Figure 3C), the treatment by apical application of antibiotics should aim to reduce *S*_a_ down below *S*^−^ to turn off the *R*-switch (Figure 7A). Once the *R*-switch is turned off, further use of antibiotics is no longer needed, as the healthy steady state with *R* = *R*_off_ is locally attractive ($S_{\text{a}}<S^{+}$) for all the parameter combinations tested (over 10, 000 simulations). The minimal treatment potency of apically applied antibiotics (minimal strength × minimal duration) to bring *S*_a_ down below *S*^−^ depends on the severity of the phenotype measured by the deviation of the high focal point from *S*^−^ (*R*^2^ = 0.46804).

When the patients are susceptible for sepsis (Figure 3D), the treatment by systemic application of antibiotics should aim to reduce *S*_v_ to avoid reaching $S_{\text{v}}^{*}$ and thereby causing invasive infection (Figure 7B). The minimal strength of systemically applied antibiotics allows the maximum of *S*_v_ to reach just below $S_{\text{v}}^{*}$, and the minimal duration of the treatment with the minimal strength corresponds to the time required for *R*(*t*) to naturally turn off by *S*_a_ reaching *S*^−^. The minimal treatment potency of systemic antibiotics to prevent sepsis depends on the severity of the phenotype measured by the time to reach $S_{\text{v}}^{*}$ in the absence of treatments.

When the patients are susceptible to the combination of sepsis and immunological scarring (Figure 3E), they require antibiotics that are strong enough to be apically applied until *S*_a_ decreases below *S*^−^ to turn off the *R*-switch (Figure 7C). Our model simulations predicted that the apical treatment is enough to prevent invasive infection for 48% of these cases, since the reduction of *S*_a_ also reduces *S*_v_, but the remaining 52% of the cases require additional application of comparatively small amounts of antibiotics directly in the blood vessel.

The distributions of the minimal treatment strengths and durations that we computationally predicted can be used as a guide to design safe and effective treatment options for the three patient cohorts. For example, our results suggest that antibiotics treatment for 20 days can prevent or revert most of immune scarring (Figure 7A) or invasive infection (Figure 7B), but that a much longer antibiotics treatment is needed for patients with a propensity for both sepsis and immune scarring (Figure 7C).

## 3. Discussion

In this paper, we have proposed the first mathematical model of *Sp* colonization of the upper airway epithelium, and demonstrated that it robustly reproduces the healthy co-existence between this bacterium and the host. Our mathematical model is a hybrid system of ODEs, describing the interactions between the bacteria, immune cells and epithelial barrier function in a mechanistic, dynamical, quantitative and integrative way.

A key element of our model to determine the healthy and pathological phenotypes is a “double switch motif” (Domínguez-Hüttinger et al., in press). The first switch describes activation of the TLR2 signaling pathway by apically located bacteria (Figure 1C). It can reflect both the resting, homeostatic *Sp*-upper airway interactions (when *R* = *R*_off_) characteristic of *Sp* as a commensal bacterium, and the transient host response to a *Sp* challenge (when *R* = *R*_on_). Failure to inactivate this *R*-switch due to impaired host-pathogen interactions, for example by weakened immune responses (Figures 4B, 5B), can have long term consequences such as immunological scarring (Figure 3C) that require treatment to resolve it. The second switch distinguishes a transient growth of *S*_v_ that can be contained without treatments (when $S_{\text{v}}\leq S_{\text{v}}^{*}$) and an invasive infection (when $S_{\text{v}}>S_{\text{v}}^{*}$) which would require a large dose of antibiotics treatments (Figure 1D). Our model simulations predict that invasive infection is developed within 36 h, in consistent with experimental observations (Andonegui et al., 2009) that were not used for development of our model. Our model analysis identified the most likely risk factors for an increased susceptibility to develop invasive infection, in response to transient *Sp* challenges (Figures 4, 5). Based on the state of this double-switch motif, we characterized four different phenotypes (Figure 3), and identified those susceptible cohorts that require specific antibiotics treatment to prevent or revert the adverse effects of a *Sp* challenge. We further used our mathematical model to calculate the minimal strengths and durations of antibiotics application to effectively treat each of these disease phenotypes (Figure 7). These results suggest that the proposed quantitative and systems-level framework of *Sp* infection can be used to design optimal and personalized treatment strategies, as it can predict the minimal application times that are required to achieve prevention or remission for individual patient cohort.

While our mathematical model was constructed based on murine and *in vitro* experiments, future calibration of the model with human data could make the proposed mathematical modeling framework directly translatable to the clinic, to help stratification of patients and identification of patient-specific optimal treatment strategies. For example, our model analysis suggested that the efficacy of bacterial killing by immune cells (Figures 4, 5) could be used as a marker to distinguish vulnerable patient cohorts who would require preventive treatments *before* the onset of sepsis. This efficacy could be determined *ex vivo*, from serum or broncheoalveolar lavage fluid extracted from patients, to predict patient-specific responses characteristic of those vulnerable patient cohorts. A similar approach to stratify patients based on measurements of isolated components of a more complex physiological system has been shown to be effective for other complex diseases (Fey et al., 2015). The computational method demonstrated in this paper could then allows us to predict the minimal strength and duration of antibiotics application for individual patient cohort and for a specific antibiotics, given the experimentally determined information on the efficacy of the antibiotics (Mandell et al., 2007; Prina et al., 2015b) and the growth rate of the pneumococcal strain in the patients' serum. Our model will also enable us to investigate and design preventive strategies by early vaccines against invasive infection in patient cohorts who are identified to be high-risk. Extension of our modeling framework to human disease will also require systematic investigation of the dose-dependent outcome of *Sp*-airway interactions (Yershov et al., 2005). Another interesting future research direction includes the assessment of the long-term effects of immunological scarring on subsequent *Sp* challenges with different amplitudes and frequencies to identify the mechanisms behind the increased risk of developing serious infections after a first bacterial challenge (Habibzay et al., 2013). Finally, extending our model to incorporate the local spread of *Sp* from the upper airway epithelium to the lung and other sites on the respiratory epithelium that are normally sterile, for example by combining our model to the model of Smith et al. (2013), could allow us to investigate the association between a dysregulated colonization of the upper airway epithelium and the development of pneumococcal pneumionia.

The results of our mathematical model of commensal bacteria infection at the upper airway epithelium shed light on the mechanism behind a loss of homeostasis caused by dysregulation of the complex interactions between epithelial surfaces and microorganisms. A key element in this control structure is a “double-switch motif,” which has been shown to govern other complex epithelial diseases, such as Atopic dermatitis (Domínguez-Hüttinger et al., in press) and cancer (Tian et al., 2013). Analysis of complex disease with a mechanistic, quantitative and systems-level framework as proposed here will help to reveal further general mechanisms underlying epithelium function in health and disease.

## 4. Methods

### 4.1. Model description

The proposed model for commensal bacterial infection describes the dynamics of bacterial load on the surface of the airway epithelium barrier, *S*_a_(*t*)[CFU/ml], infiltrated bacterial load, *S*_v_(*t*)[CFU/ml], concentrations of neutrophils and macrophages on the surface of the mucosal barrier, *N*(*t*) and *M*(*t*) [cells/ml], and the strength of barrier integrity, *B*(*t*) relative to the maximum strength, by

(1a) $$\begin{array}{l} \frac{dS_{\text{a}}(t)}{dt}=\frac{\kappa_{\text{S}}}{\mu_{\text{S}}}S_{\text{a}}(t)(1-S_{\text{a}}(t))-\frac{\theta_{\text{S}}}{1+\epsilon_{\text{SB}}B(t)}S_{\text{a}}(t)\\ \;-\phi_{\text{NS}}N(t)S_{\text{a}}(t)-\phi_{\text{MS}}M(t)S_{\text{a}}(t), \end{array}$$

(1b) $$\frac{dS_{\text{v}}(t)}{dt}=\kappa_{\text{S}}S_{\text{v}}(t)+\frac{\theta_{\text{S}}}{1+\epsilon_{\text{SB}}B(t)}S_{\text{a}}(t)-\frac{\delta_{\text{S}}}{K+S_{\text{v}}(t)}S_{\text{v}}(t),$$

(1c) $$\frac{dN(t)}{dt}=\alpha \frac{R(S_{\text{a}}(t))}{\left(1+\epsilon_{\text{NB}}B(t))(1+\epsilon_{\text{NM}}M(t)\right)}N_{\text{v}}-\delta_{\text{N}}N(t),$$

(1d) $$\frac{dM(t)}{dt}=\beta \frac{N(t)}{1+\epsilon_{\text{MB}}B(t)}M_{\text{v}}-\delta_{\text{M}}M(t),$$

(1e) $$\begin{array}{l} \frac{dB(t)}{dt}=\frac{\kappa_{\text{B}}}{1\;+\;\epsilon_{\text{BS}}R(S_{\text{a}}(t))}B(t)(\tilde{B}-B(t))\\ \;-\phi_{\text{SB}}R(S_{\text{a}}(t))B(t)-\phi_{\text{NB}}N(t)B(t). \end{array}$$

The variable *R*(*S*_a_(*t*)) denotes the *S*_*a*_-dependent TLR activation level described by a perfect switch,

(2) $$R(S_{\text{a}}(t))=\left\{ \begin{array}{l} R_{\text{off}}\text{ if }S_{\text{a}}(t)<S^{-}\text{ or }\{S^{-}\text{ ​​}\leq S_{\text{a}}(t)<S^{+}\text{ and}\\ \;R(S_{\text{a}}(t^{-}))=R_{\text{off}}\},\\ R_{\text{on}}\text{ if }S_{\text{a}}(t)\geq S^{+}\text{ or }\{S^{-}\text{ ​​}\leq S_{\text{a}}(t)<S^{+}\text{ and}\\ \;R(S_{\text{a}}(t^{-}))=R_{\text{on}}\}, \end{array}\right.$$

where *t*^−^ is a time slightly before the time *t*. The dynamics of the TLR activity stabilizes within hours (Filewod et al., 2009; Witt et al., 2009; Hoffman et al., 2015).

The growth of the bacterial load on the apical side of the barrier, *S*_a_, is modeled by a logistic equation (Smith et al., 2011), where the growth rate is limited by a carrying capacity (saturation term μ_S_) that reflects the limited availability of nutrients in the epithelial lumen (Burnaugh et al., 2008). *S*_a_ is eradicated by immune cells, *N* and *M*, and transmigrates to the basal side of the epithelial barrier. The transmigrated bacteria, *S*_v_, is assumed to grow exponentially in the blood vessel with abundant nutrients, but is killed by resident immune cells. The capacity to contain *S*_v_ is described by the saturated degradation of *S*_v_, leading to the complete decay of *S*_v_ if it is below the threshold $S_{\text{v}}^{*}$, which corresponds to the unstable steady state of the ODE for *S*_v_ when $S_{\text{a}}=S^{-}$.

Recruitment of neutrophils (*N*) and macrophages (*M*) to the site of infection from their respective pool in the blood vessel (*N*_v_) and the airway tissues (*M*_v_) is inhibited by the epithelium barrier integrity (*B*), and is enhanced by the TLR activation (*R*) and the recruited neutrophils, respectively. Recruitment of *N* is further inhibited by *M*. *N* and *M* decay with a respective constant decay rate, as *de novo* production of the immune cells does not occur outside the bone marrow (Tak et al., 2013) and they do not divide in the epithelial tissue.

The self-recovery of the mucosal barrier to its homeostatic level (Nusrat et al., 1997; Coyne et al., 2002; Heijink et al., 2012) is modeled in a phenomenological manner, with the recovery rate being compromised by a decreased gene expression of epithelial cell differentiation markers (Clarke et al., 2011) induced by TLR activation. The barrier is directly damaged by transmigrating neutrophils and by proteases that are activated via TLR signaling (Chun and Prince, 2009). The switch-like activation of the TLR signaling is triggered by apically located bacteria, and is modeled by a phenomenological representation (Mochan et al., 2014; Domínguez-Hüttinger et al., in press). For simplicity, the inhibition by *x* is modeled phenomenologically by $\frac{1}{1\;+\;x}$.

### 4.2. Numerical integration of the hybrid model

All the numerical model analysis was conducted using MATLAB version R2014a (The MathWorks, Inc., Natick, MA, USA). Numerical integration was conducted by ode15s from the initial conditions corresponding to a transient *Sp* challenges with $S_{\text{a}}\left(0\right)=10^{7}$ CFU/ml, *S*_v_(0) = 0 CFU/ml, *N*(0) = 0 cells/ml, *M*(0) = 10 cells/ml, and *B*(0) = 1, with *R*(0) = 1, as in the experiments in Zhang et al. (2009). The switch-dependent governing equation was chosen by the event-location function.

### 4.3. Modeling antibiotics—calculation of minimal strength and minimal duration of antibiotics treatment

We model the effects of bactericidal antibiotics, such as penicillin, ceftriaxone and amoxilin, which are commonly prescribed to treat pneumococcal infection (Mandell et al., 2007; Prina et al., 2015b) by $\frac{dS_{\text{v}}}{dt}=-VS_{\text{v}}\left(t\right)$ (systemic application of antibiotics) and $\frac{dS_{\text{a}}}{dt}=-AS_{\text{a}}\left(t\right)$ (apical application of antibiotics), where *V* and *A* represent a constant strength of antibiotics that kill *S*_v_(*t*) and *S*_a_(*t*), respectively. The strength of the antibiotics (with a unit of 1/*h*) is described by *V* = *E*_V_*D*_V_ or *A* = *E*_A_*D*_A_, where *E*_V_ and *E*_A_ is the antibiotics killing efficacy and *D*_V_ and *D*_A_ is the amount applied, and can be chosen in the clinic by either selecting an antibiotic with a particular killing efficacy and/or adjusting the dose administrated. The killing efficacy of an antibiotic over a specific bacterial strain is commonly evaluated by the Minimal Inhibitory Concentration (MIC), the lowest concentration of antibiotics that will inhibit the visible growth of a bacterium after overnight incubation in a kinetic growth assay. From such experimental information, the antibiotics efficacy in our model, *E*_A_ (and *E*_V_) can be calculated as $E_{\text{A}}=\frac{\kappa_{\text{S}}}{\mu_{\text{S}}}\left(1-S_{\text{MIC}}\left(t\right)\right)\times \frac{1}{D_{\text{A}}^{\text{MIC}}}$, where *S*_MIC_(*t*) is the concentration of *Sp* exposed to an antibiotics dose of $D_{\text{A}}^{\text{MIC}}$ = MIC, and hence does not increase further. This expression is obtained from the steady state equation $\frac{dS_{\text{MIC}}\left(t\right)}{dt}=\kappa_{\text{S}}\mu_{\text{S}}\left(1-S_{\text{MIC}}\left(t\right)\right)-E_{\text{A}}D_{\text{A}}^{\text{MIC}}S_{\text{MIC}}\left(t\right)=0$, which holds for the value of *S*_MIC_(1*day*) = *S*_a_ in these experiments.

Minimal strength of antibiotics treatment to achieve remission was determined by checking whether $S_{\text{v}}\left(t\right)<S_{\text{v}}^{*}$ or $S_{\text{a}}\left(t\right)<S^{-}$ is achieved while gradually increasing *V* or *A*, respectively, by an increment of 0.01. The minimal duration of treatments corresponds to the time required to achieve $S_{\text{a}}=S^{-}$. The minimal antibiotics treatment regimens to revert immune scarring without invasive infection (Figure 7A) were calculated under the assumptions that the treatment starts when *S*_a_ reached its steady state, after a transient *S*_a_ challenge that was modeled with initial conditions of $S_{\text{a}}\left(0\right)=10^{7}$ CFU/ml, *S*_v_(0) = 0 CFU/ml, *N*(0) = 0 cells/ml, *M*(0) = 10 cells/ml, and *B*(0) = 1, with *R*(0) = 1. For the regimens to prevent invasive infection (Figure 7B), as well as to reverse immunological scarring and to prevent sepsis (Figure 7C), we assumed that the treatment starts at the time of the *S*_a_ challenge.

### 4.4. Robustness analysis and parameter sensitivity analysis

We varied all the model parameters over one order of magnitude (0.1 - 10 times) around the nominal values, and the initial conditions *N*(0), *M*(0) and *B*(0) within the intervals [0 1,000], [0 100] and [0 1], respectively. Robustness of the healthy behavior was tested by simultaneously varying all the parameters which were sampled from uniform distributions for 10, 000 iterations. The global parameter sensitivity was evaluated by 10, 000 iterations using the Global Sensitivity Analysis Toolbox for Matlab (Cannavó, 2012), with respect to the final concentrations of *S*_v_ and *R* at 7 days post *Sp* challenge with $S_{\text{a}}\left(0\right)=10^{7}$ CFU/ml.

### 4.5. Focal point analysis

We conducted a focal point analysis to determine the long-term behavior of the model in absence of a *Sp* challenge. Following the methodology in Oyarzún et al. (2012), we considered two subsystems for Equation (1), defined by fixing *R* to either *R* = *R*_off_ or to *R* = *R*_on_, and evaluated the local stability of their steady states.

## Ethics statement

Blood used in pneumococcal growth assays was obtained from healthy volunteers who had given written consent. Ethical approval for this work was obtained from the Tissue Management Committee of the ICHTB (Project: R14053, ICHTB HTA license: 12275, REC Wales approval: 12/WA/0196). Tissue samples were provided by the Imperial College Healthcare NHS Trust Tissue Bank. Other investigators may have received samples from these same tissues. The research was supported by the National Institute for Health research (NIHR) Biomedical Research Centre based at Imperial College Healthcare NHS Trust and Imperial College London. The views expressed are those of the author(s) and not necessarily those of the NHS, NIHR, or the Department of Health.

## Author contributions

ED, TC, NB, and RT designed the research, developed the mathematical model and analyzed data. ED performed the computational experiments. TC performed the *Sp* growth experiments. ED, TC, and RT wrote the paper.

## Funding

ED acknowledges funding from the Mexican Council for Science and Technology (CONACyT, Ph.D. scholarship 212800) and from the National Autonomous University of Mexico (UNAM, postdoctoral scholarship). TC is a Sir Henry Dale Fellow jointly funded by the Wellcome Trust and Royal Society (grant No. 107660/Z/15/Z). RT acknowledges EPSRC Career Acceleration Fellowship (EP/G007446/1).

### Conflict of interest statement

The authors declare that the research was conducted in the absence of any commercial or financial relationships that could be construed as a potential conflict of interest.
