# Supplementary material for: Mathematical Modeling of Streptococcus pneumoniae Colonization, Invasive Infection and Treatment
Source: Front Physiol. 2017 Mar 2;8:115. doi: 10.3389/fphys.2017.00115 (PMC5332394; doi:10.3389/fphys.2017.00115)
Supplement: Supplementary file 1 [file Presentation1.PDF]

frontiers

Supplementary Material: Mathematical modelling of colonization, invasive infection and treatment of *Streptococcus pneumoniae*

Elisa Domínguez-Hüttinger [1,2], **Neville J. Boon** [1], **Thomas Clarke** [3] **and Reiko J. Tanaka** [1,*]

*Correspondence:
Reiko J. Tanaka
Department of Bioengineering, Imperial College London, London SW7 2AZ, United Kingdom, r.tanaka@imperial.ac.uk

Elisa Domínguez-Hüttinger
Instituto de Ecología, Universidad Nacional Autónoma de México, Mexico City 04510, Mexico, elisa.dominguez@mail.ecologia.unam.mx

PARAMETER ESTIMATION

We derived the 23 model parameters in three steps:

1. Direct estimation of four parameters,
2. Indirect estimation of other nine parameters, by fitting the model results to five datasets from *in vitro* studies simultaneously, while fixing the four parameter values obtained above.
3. Indirect estimation of the remaining 10 parameters, by fitting the model results to two datasets from *in vivo* studies simultaneously, while fixing the 13 parameter values obtained above.

We describe in the followings the details of the methods and datasets used to estimate the parameters.

Direct parameter estimation

N_v and δ_N

Derived as in Tanaka et al. (2015).

κ_B

In Coyne et al. (2002), a monolayer of human airway epithelial cells were incubated with $10\mathrm{ng\ ml}^{-1}$ TNFα and $100\mathrm{ng\ ml}^{-1}$ INFγ for 72 hours, before the cytokines were washed away and the transepithelial resistance was monitored for 24 hours. The data in Fig 1B in Coyne et al. (2002), $B(0) = 100$, $B(4) = 200$, $B(12) = 700$ and $B(24) = 800$, is normalised by its maximum control value $B_\mathrm{max} = 1000$ to be $\tilde{B}(0) = 0.1$, $\tilde{B}(4) = 0.2$, $\tilde{B}(12) = 0.7$ and $\tilde{B}(24) = 0.8$. We fitted this data to the solution $B(t) = 1 + \mathrm{e}^{-\kappa_\mathrm{B} t}(B(0) - 1)$ of the equation $\dot{B}(t) = \kappa_\mathrm{B}(1 - B(t))$ to derive $\kappa_\mathrm{B} = 0.046\ \mathrm{h}^{-1}$ (Fig. S1).

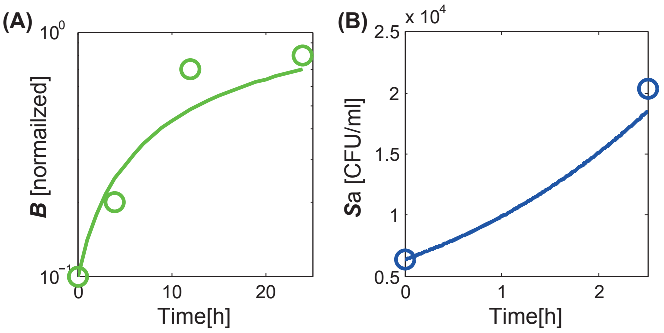

Supplementary Figure 1. Direct estimation of (A) κ_{B} and (B) κ_{S} by fitting of the model simulation (lines) to experimental data (circles).

κ_{S}

Hathaway et al. (2012) measured the amount of *Streptococcus pneumoniae* (in OD 450) at 2.5 and 5 h (Figure 1B). Fitting the data $S_{\mathrm{a}}(2.5) = 0.1$, and $S_{\mathrm{a}}(5) = 0.32$ to $\dot{S}_a = \kappa_{\mathrm{S}} S_a$ without saturation results in $\kappa_{\mathrm{S}} = 0.48\ \mathrm{h}^{-1}$ (Fig. S1).

Indirect parameter estimation using data from *in vitro* studies

While fixing the values of the four parameters obtained above, we derived the nominal values for other nine parameters by minimising a single-valued cost function defined as a sum of least-squares for the simultaneous fit to five datasets from *in vitro* studies as detailed below. We assumed the equal uncertainties for all the data used. The results of the fitting are shown in Fig.S2.

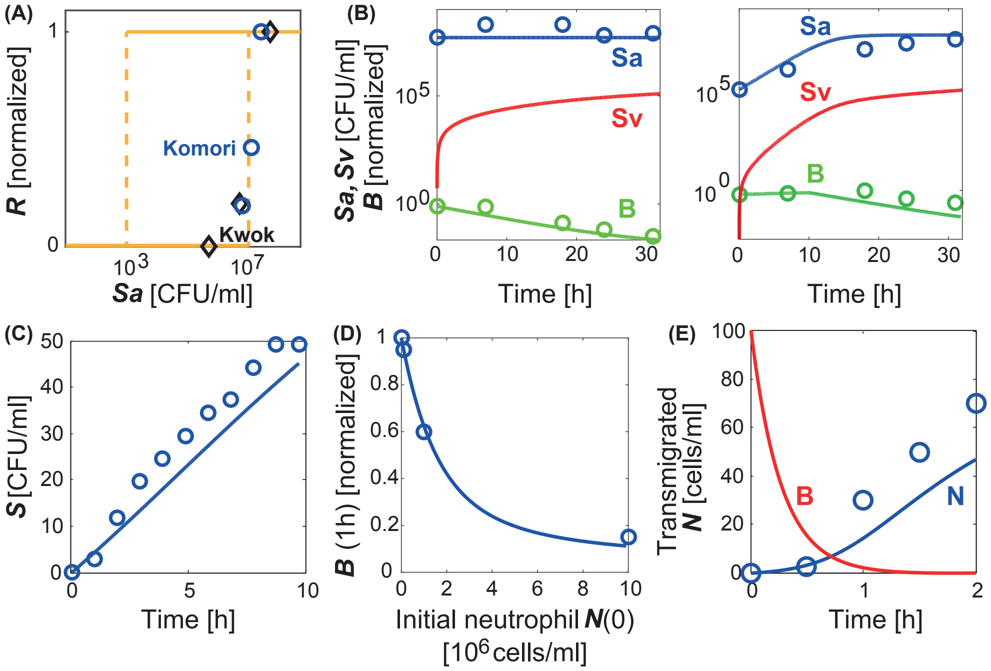

Supplementary Figure 2. Indirect estimation of (A) S^{+} and S^{-}, (B, C) θ_{S}, ϵ_{BS}, ϵ_{SB} and ϕ_{SB}, (D, E) ϵ_{NB} and ϕ_{NB}, by simultaneous fitting of the simulation results (lines) to the five datasets (circles) from *in vitro* studies.

S^+ and S^-

We fitted data that measured the output level of TLR pathway activity in response to a bacteria-derived ligand (peptidoglycan) to the R-switch in our model (Eq. 2), and derived $S^+ = 10^7$ and $S^- = 10^3$ CFU/ml (Fig. S2(A)). The data used were the levels of NFκB activation on the middle ear epithelial cells in Komori et al. (2011) (reproduced in Table S1) and the levels of IL-23 mRNA on peripherial blood mononuclear cells Kwok et al. (2012) (reproduced in Table S2). We assumed $R_{\rm off} = 0$, $R_{\rm on} = 1$ and that 1μg of peptidoglycan correspond to 5×10^7 CFU of *S. pneumoniae* ($S_{\rm a}$)(Travassos et al., 2004).

Supplementary Table 1. Data from Komori et al. (2011) Fig 3A

Peptidoglican μ g/ml	0	0.125	0.25	0.5
NFκB activity (fold expression)	1	1.25	1.6	2.3

Supplementary Table 2. Data from Kwok et al. (2012) Fig 3A

Peptidoglican ng/ml	0	0.01	0.1	1
IL-23 mRNA (fold expression)	1	1.25	2.5	6

$\theta_{\rm S}, \epsilon_{\rm BS}, \epsilon_{\rm SB}, \phi_{\rm SB}$

We fitted the data from bacteria-primary culture system in Lagrou et al. (2003) (Fig. S2(B)) and bacteria-monolayer culture system in Attali et al. (2008) (Fig. S2(C)) to the model of bacteria-barrier system (a subsystem of our proposed model) described by

$$\frac{dS_{\rm a}}{dt} = \kappa_{\rm S} S_{\rm a} - \frac{\theta_{\rm S}}{\epsilon_{\rm SB} B + 1} S_{\rm a}, \tag{S1a}$$

$$\frac{dS_{\rm v}}{dt} = \kappa_{\rm S} S_{\rm v} + \frac{\theta_{\rm S}}{\epsilon_{\rm SB} B + 1} S_{\rm a}, \tag{S1b}$$

$$\frac{dB}{dt} = \frac{\kappa_{\rm B}(1-B)}{1 + \epsilon_{\rm BS} R(S'_{\rm a})} - \phi_{\rm SB} R(S'_{\rm a}) B, \tag{S1c}$$

and derived the nominal values for $\theta_{\rm S}, \epsilon_{\rm BS}, \epsilon_{\rm SB}$, $\phi_{\rm SB}$, and $S_{\rm a}(0)$. Killing of $S_{\rm v}$ is not included in the model as no immune cells to kill the bacteria exist in the transwell culture. The new variable $S'_{\rm a}$ is the concentration of bacteria that interacts with the barrier at transwell. We assumed $S'_{\rm a} = 20 S_{\rm a}$, since the area of the monolayer used in the transwell experiment ($30{\rm cm}^2$) is 20 times smaller than the area of mouse lung epithelial surface (for which the model was developed) (Wangensteen, 1994).

The data from Lagrou et al. (2003) is the dynamics of the barrier integrity of a primary culture of human nasopharyngeal epithelium and of *S. pneumoniae* (reproduced in Table S3). To fit the model of bacteria-barrier system (Eqs. S1) to this data, we assumed a carrying capacity $\mu_{\rm S} = 0.5 \times 10^8$ CFU/ml, which corresponds to the level where $S_{\rm a}$ saturates under the nutrient-rich condition of this *in vitro* experiment.

The data from Attali et al. (2008) is the percentage of infiltration of *S. pneumoniae* through a monolayer of airway epithelial cells (A549), corresponding to $\frac{S_{\rm v}}{S_{\rm a}+S_{\rm v}}$, measured by florescence of cells (reproduced in Table S4). The initial concentration of apically located bacteria was $S_{\rm a}(0) = 5 \times 10^4 \times 10^5$ CFU/ml.

Supplementary Table 3. Data from Lagrou et al. (2003) Fig 1 with different $S_{\rm a}(0)$

Time (hours)	0	7	18	24	31
Fig. 1a					
TEER ($\Omega{\rm cm}^2$)	100	113	150	60	40
S_a (10^5 cells/ml)	1	10	100	199	316
Fig. 1b					
TEER ($\Omega{\rm cm}^2$)	120	110	20	10	5
S_a (10^5 cells/ml)	0.5 $\times 10^3$	2 $\times 10^3$	2 $\times 10^3$	0.6 $\times 10^3$	0.8 $\times 10^3$

Supplementary Table 4. Data from Attali et al. (2008) Fig. 5b

Time (hours)	0	1	2	3	4	5	6	7	8	9	10
% of infiltrated bacteria	0	3	12	20	25	30	35	38	45	50	50

The nominal values for $\theta_{\rm S}$ and $\epsilon_{\rm SB}$ shown in Table 1 are 1000 times lower and 0.5 times higher, respectively, of the values obtained from the fitting to the data from Attali et al. (2008). These scaling factors were introduced to account for a weaker barrier in the transwell experiments (Wilson et al., 1996), compared to the *in vivo* counterpart, due to the absence of the endothelium and the mucus layer that increase the barrier strength.

$\epsilon_{\rm NB}$ and $\phi_{\rm NB}$

We fitted the data from neutrophil-monolayer culture system in Nash et al. (1987) (Fig.S2(D)) and in Chin et al. (2008) (Fig.S2(E)) to the model of neutrophil-PAMPS-barrier system (a subsystem of our proposed model) described by

$$\frac{dN}{dt} = R(\hat{S})N_{\rm b}\frac{\theta_{\rm N}}{\epsilon_{\rm NB}B+1} - \delta_{\rm N}N, \tag{S2a}$$

$$\frac{dN_{\rm b}}{dt} = -R(\hat{S})N_{\rm b}\frac{\theta_{\rm N}}{\epsilon_{\rm NB}B+1} - \delta_{\rm N}N, \tag{S2b}$$

$$\frac{dB}{dt} = \kappa_{\rm B}(1-B) - \phi'_{NB}NB, \tag{S2c}$$

$$\frac{d\hat{S}}{dt} = -\frac{\hat{\theta}_{\hat{S}}}{\hat{\epsilon}_{\rm SB}B+1}\hat{S}. \tag{S2d}$$

The variables $\hat{S}(t)$ and $N_{\rm b}$ in the model represent the dynamics of the PAMPs and the number of neutrophils added to the basolateral chamber of the transwell, respectively. $\hat{S}(t)$ corresponds to the concentration of a biochemical molecule that is affected only by the infiltration through the barrier. To fit the neutrophil-PAMPS-barrier system to the experimental *in vitro* data of Nash et al. (1987) and Chin et al. (2008), we introduced new parameters: the *in vitro* transmigration rate of the neutrophils ($\theta_N = 0.1$ h), and the transmigration rate of the PAMPs ($\hat{\theta}_{\hat{S}} = 0.01$ h). The parameter $\phi'_{\rm NB}$ is the rate of barrier damage by the basal neutrophils at transwell concentrations, and is assumed to satisfy $\phi'_{\rm NB} = 20\phi_{\rm NB}$, since the area of a

monolayer experiment is 20 times smaller than that of the mouse lung epithelial surface (Wangensteen, 1994).

The data from Nash et al. (1987) (reproduced in Table S5) is a dose-response curve obtained by measuring the concentrations of transmigrated neutrophils and the integrity (resistance) of T84 epithelial (inverted) monolayer (human colorectal adenocarcinoma, a transformed epithelial cell line) at 1 hour post-treatment by 10^{-7}M fMLP, which corresponds to PAMPs.

Supplementary Table 5. Data from Nash et al. (1987) Fig 4

$N_{\mathrm{b}}(0)$ (cells $\times 10^5$/ml)	0.01	1	10	100
$B(1h)$ (100-percentage of resistance fall from baseline)/100)	1	.95	0.6	0.15

The data from Chin et al. (2008) (reproduced in Table S6) is the percentage of applied neutrophils that have transmigrated from the basal to the apical side of the epithelial monolayer, after applying 50 cells $\times 10^5$/ml of neutrophils.

Supplementary Table 6. Data from Chin et al. (2008) Fig 2b

Time (hours)	0	0.5	1	1.5	2
% of transmigrated N	0	2.5	30	50	70

Indirect parameter estimation using data from *in vivo* studies

While fixing the values of the 13 parameters obtained above, we derived nominal values for the remaining 10 parameters by minimising a single-valued cost function defined as a sum of least-squares for the simultaneous fit to the two datasets from *in vivo* mice studies as detailed below. We assumed the equal uncertainties for all the data used. The results of the fitting are shown in Fig. 2 in the main text.

$\mu_{\mathrm{S}}, \phi_{\mathrm{NS}}, \alpha, \epsilon_{\mathrm{NM}}$, β M_{v}, θ_{S} and δ_{M}

We fitted the mouse experiment data from Zhang et al. (2009) to our full model (Eqs. 1, 2) to derive $\mu_{\mathrm{S}}, \phi_{\mathrm{NS}}, \alpha, \epsilon_{\mathrm{NM}}$, β M_{v}, θ_{S} and δ_{M}. We re-estimated the value of θ_S (the rate of bacterial transmigration through the barrier), which was already estimated by fitting to the data from *in vitro* studies. It is because the invasion of the apically located bacteria to the blood stream is restricted *in vivo* not only by the tight junctions of the airway epithelial cells, but also by other factors such as the mucus layer (Johansson et al., 2010) and the endothelium (Wilson et al., 1996). This re-estimation identified scaling factors that explain the difference in those parameter values fitted from *in vitro* and *in vivo* studies. The data used (reproduced in Table S7) are the luminal concentrations of bacteria (S_{a}), neutrophils (N) and macrophages (M) of the nasal fluid of sacrificed mice (extracted by lavage) after the nares of mice (C57BL/6, wt and $TLR2^{-/-}$) were instilled with 10^7 CFU of *S. pneumoniae* (strain P1121, derived from the nasopharinx of a human subject).

Supplementary Table 7. Data from Zhang et al. (2009) Figs 1a-c and 6

Time (days)	0	1	3	3.5	5	7	21
S_a (log CFU/animal)	n/a	4.5	4	4	4.2	3.8	1.6
N (cells/animal)	0	5	200	300	50	80	0
M (cells/animal)	10	20	50	75	65	80	60

K and δ_S

We fitted the mouse experimental data from Benton et al. (1997) and our own experiment described below, to the subsystem of S_v dynamics described by

$$\frac{dS_v}{dt} = \kappa_S S_v - \frac{\delta_S}{K + S_v} S_v \tag{S3}$$

and derived the critical threshold $S_v^* = 10^3$ (Fig S3), from which nominal values for K and δ_S were derived.

The data from Benton et al. (1997) (reproduced in Table S8) is the concentration of *S. pneumoniae* (strain D39 type 2) that survived in the blood after an intravenous challenge (0.2 ml of bacteria at 500×10^5 CFU/ml, thus $0.2 \times 500 \times 10^5 = 10^7$ CFU). The bacterial concentrations were calculated using the estimated volume of circulating blood in a mouse, 1.4625 ml (Tanaka et al., 2015). The data show that the bacteria grow exponentially from $S_v(0) = 10^{3.75}$ CFU/ml.

Supplementary Table 8. Data from Benton et al. (1997) Fig 1a

Time (hours)	0	8	15	24	30
S_v (log CFU/ml)	3.75	5	6.67	7.5	11.67

The data from our own experiment is the dynamics of bacterial load after inoculating 10^2 CFU of *S. pneumoniae* (strain D39) into 1ml of human blood (Table S9, mean of two replicates). *S. pneumoniae* was grown in tryptic soy broth to mid-log phase, washed with PBS and then re-suspended in fresh whole human blood. Blood was obtained from healthy volunteers (see ethics for consent information). Growth of *S. pneumoniae* in whole blood was determined over four hours using a range of initial bacterial colony forming units. The data demonstrates a rapid clearance of the initial bacteria if the concentration is below 10^2 CFU/ml. We assumed that the value for δ_S is 3 times higher when fitting to the human data, due to the stronger immune response in human than in mice.

Supplementary Table 9. Data from our own experiment

Time (hours)	0	0.5	1	2	4
S_v (log CFU/ml)	200	300	125	5	0

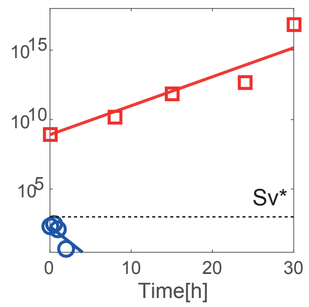

Supplementary Figure 3. Indirect estimation of the nominal values for the remaining 10 parameters (that were estimated neither directly nor fitting to the data from *in vitro* studies) by fitting of our model to the data from Benton et al. (1997) (red squares) and to our data (blue circles) to estimate S_v^*

REFERENCES

Tanaka RJ, Boon NJ, Vrcelj K, Nguyen A, Vinci C, Armstrong-James D, et al. In silico modeling of spore inhalation reveals fungal persistence following low dose exposure. *Sci. Rep.* **5** (2015) 13958. doi:10.1038/srep13958.

Coyne CB, Vanhook MK, Gambling TM, Johnny L, Boucher RC, Johnson LG, et al. Regulation of Airway Tight Junctions by Proinflammatory Cytokines. *Mol. Biol. Cell* **13** (2002) 3218–3234. doi:10.1091/mbc. E02.

Hathaway LJ, Brugger SD, Morand B, Bangert M, Rotzetter JU, Hauser C, et al. Capsule type of Streptococcus pneumoniae determines growth phenotype. *PLoS Pathog.* **8** (2012). doi:10.1371/journal. ppat.1002574.

Komori M, Nakamura Y, Ping J, Feng L, Toyama K, Kim Y, et al. Receptor 2 in the Mouse Middle Ear Epithelial Cells. *Pediatr. Res.* **69** (2011) 101–105.

Kwok SK, Cho ML, Her YM, Oh HJ, Park MK, Lee SY, et al. TLR2 ligation induces the production of IL-23/IL-17 via IL-6, STAT3 and NF-kB pathway in patients with primary Sjogren's syndrome. *Arthritis Res. Ther.* **14** (2012) R64. doi:10.1186/ar3780.

Travassos LH, Girardin SE, Philpott DJ, Blanot D, Nahori MA, Werts C, et al. Toll-like receptor 2-dependent bacterial sensing does not occur via peptidoglycan recognition. *EMBO Rep.* **5** (2004) 1000–6. doi:10.1038/sj.embor.7400248.

Lagrou K, Peetermans WE, Verhaegen J, Jorissen M, Van Eldere J. Disruption of nasopharyngeal epithelium by pneumococci is density-linked. *Eur. J. Clin. Invest.* **33** (2003) 340–345.

Attali C, Durmort C, Vernet T, Di Guilmi AM. The interaction of Streptococcus pneumoniae with plasmin mediates transmigration across endothelial and epithelial monolayers by intercellular junction cleavage. *Infect. Immun.* **76** (2008) 5350–6. doi:10.1128/IAI.00184-08.

Wangensteen O. Fluid and solute transport in the airspaces of the lung. Effros R, Chang H, editors, *Fluid solute Transp. airspaces lung*, chap. Nonselecti (1994), 374–397.

Nash S, Stafford J, Madara JL. Effects of Polymorphonuclear Leukocyte Transmigration of Cultured Intestinal Epithelial Monolayers. *J. Clin. Invest.* **80** (1987) 1104–1113.

Chin AC, Lee WY, Nusrat A, Vergnolle N, Parkos Ca. Neutrophil-mediated activation of epithelial protease-activated receptors-1 and -2 regulates barrier function and transepithelial migration. *J. Immunol.* **181** (2008) 5702–10.

Zhang Z, Clarke TB, Weiser JN. Cellular effectors mediating Th17-dependent clearance of pneumococcal colonization in mice. *J. Clin. Invest.* **119** (2009) 1899–1909. doi:10.1172/JCI36731.Moreover.

Johansson MEV, Gustafsson JK, Sjöberg KE, Petersson J, Holm L, Sjövall H, et al. Bacteria penetrate the inner mucus layer before inflammation in the dextran sulfate colitis model. *PLoS One* **5** (2010). doi:10.1371/journal.pone.0012238.

Wilson R, Dowling R, aD Jackson. The biology of bacterial colonization and invasion of the respiratory mucosa. *Eur. Respir. J.* **9** (1996) 1523–1530. doi:10.1183/09031936.96.09071523.

Benton KA, Paton JC, Briles DE. Differences in virulence for mice among Streptococcus pneumoniae strains of capsular types 2, 3, 4, 5, and 6 are not attributable to differences in pneumolysin production. *Infect. Immun.* **65** (1997) 1237–1244.
